# Topographic Relationship with a Retinal Nerve Fiber Layer Defect Differs between *β*-Zone and *γ*-Zone Parapapillary Atrophy

**DOI:** 10.1155/2020/6279689

**Published:** 2020-08-21

**Authors:** Eun Hye Jung, Eun Ji Lee, Tae-Woo Kim

**Affiliations:** ^1^Department of Ophthalmology, Seoul National University College of Medicine, Seoul National University Bundang Hospital, Seongnam, Republic of Korea; ^2^Department of Ophthalmology, Nowon Eulji Medical Center, Eulji University, Seoul, Republic of Korea

## Abstract

**Introduction:**

*γ*-Zone parapapillary atrophy (PPA), an associated feature in myopic tilted optic disc, is considered to be relevant with glaucomatous optic nerve damage in myopic eyes. This study determines the topographic relationship of *γ*-zone PPA with a retinal nerve fiber layer defect.

**Purpose:**

To determine the topographic relationship of *γ*-zone PPA with a RNFL defect and to compare it with that of *β*-zone PPA.

**Design:**

Cross-sectional, observational study. *Participants*. Eighty-nine eyes from 89 patients with primary open-angle glaucoma who had *β*-zone PPA (*n* = 49) or *γ*-zone PPA (*n* = 40) and a single localized RNFL defect.

**Methods:**

PPA was classified according to the presence or absence of Bruch's membrane on the PPA bed in spectral-domain optical coherence tomography. The angular location of the point of maximum radial extent of PPA (PMRE) and the RNFL defect was measured with the fovea-disc axis set at 0° in color and red-free fundus photographs. *Main Outcome Measures*. Angular distance between the RNFL defect and the PMRE.

**Results:**

There was no significant intergroup difference in the extent of the RNFL defect (*P*=0.920). The angular distance between the RNFL defect and the PMRE was significantly greater in *γ*-zone than *β*-zone PPA (26.49 ± 17.27° vs. 60.31 ± 17.12°, *P* < 0.001). The angular location of the PMRE was significantly correlated with the location of the RNFL defect in the *β*-zone group (*r* = 0.822, *P* < 0.001) but not in the *γ*-zone group. The RNFL defect was mostly located near the edge of *γ*-zone PPA in the *γ*-zone group (10.56 ± 9.47°).

**Conclusions:**

An RNFL defect was observed near the edge of PPA in eyes with *γ*-zone PPA, in contrast to it being close to the PMRE in eyes with *β*-zone PPA.

## 1. Introduction

Parapapillary atrophy (PPA) has been classified into peripheral *α*-zone and central *β*-zone. Studies have shown that *β*-zone PPA is a risk factor for the development [1] and progression [2, 3] of glaucoma. Based on the findings of both histologic and angiographic studies, vascular compromise has been proposed as being responsible for the association of PPA with glaucoma.

Recent studies have further classified *β*-zone PPA based on the existence of Bruch's membrane (BM) on PPA bed [4]. PPA with BM was found to be associated with old age, whereas PPA without BM was often found in young patients and associated with a long axial length and externally oblique border tissue of Elschnig [5]. These findings suggest that PPA with BM is an age-related atrophic change, while PPA without BM is derived from scleral stretching arising from the axial elongation of the eyeball. Based on similar findings, Jonas et al. [6] named PPA without BM as *γ*-zone PPA.

Cho and Park demonstrated that the location of RNFL defect in *β*-zone PPA is topographically correlated with the point of maximum radial extent of PPA (PMRE) [7]. However, this study defined *β*-zone PPA based on the conventional concept without consideration of the *γ*-zone. Given the different pathogeneses of *β*-zone and *γ*-zone PPA, we hypothesized that the topographic relationship with the RNFL defect differs between *β*-zone and *γ*-zone PPA. The comparison of the relationship of these two types of PPA with RNFL defects may expand our understanding of the pathogenic relevance of PPA to glaucoma development and progression. In particular, this comparison may provide an implication on the characteristics or biomechanics of optic nerve damage in myopic tilted eyes because *γ*-zone PPA is an associated feature of myopic tilted disc [5, 8, 9].

The purpose of this study was to compare the topographic relationship of an RNFL defect and the location of the PMRE between *β*-zone and *γ*-zone PPA.

## 2. Patients and Methods

This investigation was based on the Investigating Glaucoma Progression Study (IGPS), which is an ongoing prospective study being performed in the Glaucoma Clinic of Seoul National University Bundang Hospital. These studies were approved by the Institutional Review Board of Seoul National University Bundang Hospital and followed the tenets of the Declaration of Helsinki. Written informed consent was obtained from all patients.

### 2.1. Study Subjects

Each subject who was enrolled in the IGPS received a comprehensive ophthalmic examination that included visual acuity assessment, Goldmann applanation tonometry, slit-lamp biomicroscopy, gonioscopy, refraction tests, stereo disc photograph, red-free fundus photograph, spectral-domain (SD) optical coherence tomography (OCT) (Spectralis, Heidelberg Engineering, Heidelberg, Germany), central corneal thickness measurement (Orbscan II, Bausch & Lomb Surgical, Rochester, NY, USA), axial length measurement (IOL Master version 5, Carl Zeiss Meditec, Dublin, CA, USA), and 24-2 Swedish interactive threshold algorithm standard of the Humphrey Field Analyzer II 750 (Carl Zeiss Meditec). To be included in either study, subjects were required to have a best-corrected visual acuity of ≥20/40, a spherical refraction of −8.0 to +3.0 diopters, and a cylinder correction of −3.0 to +3.0 diopters. Those with a history of ocular surgery other than cataract extraction and glaucoma surgery, and intraocular disease (e.g., diabetic retinopathy or retinal vein occlusion) or neurologic disease (e.g., pituitary tumor) that could cause visual field loss were excluded. When both eyes of a subject were eligible, one eye was randomly chosen for data analysis.

To be included in the present study, subjects were required to have primary open-angle glaucoma (POAG), a single localized RNFL defect with an angular extent of 15–60° (as assessed in red-free fundus photography), and *β*-zone PPA (based on the conventional classification [10]) visible in stereo disc photography with a width of 200 *μ*m or more on at least one radial SD-OCT B-scan as measured by the built-in caliper tool of the Spectralis OCT device (see below). POAG was defined as a glaucomatous optic nerve change (e.g., focal thinning, notching, and an RNFL defect), a glaucomatous visual field defect, and an open iridocorneal angle. A glaucomatous visual field defect was defined as (1) outside the normal limit in the glaucoma hemifield test or (2) three abnormal points, with *P* <5% probability of being normal, one with *P* <1% by pattern deviation; or (3) a pattern standard deviation (PSD) of <5% confirmed in two consecutive reliable tests (fixation loss rate ≤20% and false-positive and false-negative error rates ≤25%). Eyes with degenerative myopia and those with poor-quality SD-OCT images were excluded.

### 2.2. Identification of RNFL Defects and *β*-Zone PPA

Stereo disc photographs and red-free fundus photographs were obtained using a digital camera (EOS D60, Canon, Utsunomiyashi, Tochigiken, Japan) after dilating the pupil. A localized RNFL defect was defined on a red-free fundus photograph as a well-outlined, dark, wedge-shaped area, with the end of the dark area touching the optic disc border [11].

*β*-Zone PPA was defined as a region of chorioretinal atrophy adjacent to the optic disc, with visible sclera and choroidal vessels in stereo disc photographs [10]. At this step, PPA was not separated into *β*-zone and *γ*-zone PPA.

### 2.3. Spectralis OCT Imaging of the Parapapillary Area

The optic disc including the parapapillary area was scanned by the Spectralis OCT device using the enhanced-depth imaging technique [12]. Radial B-scans were obtained using 24 radial lines centered on the optic disc. Each section had 30 OCT frames averaged and was 1024 pixels (6.1 mm) in length. The structure of *β*-zone PPA determined in the fundus imaging was evaluated using the Spectralis viewer (Heidelberg Eye Explorer software version 1.7.0.0, Heidelberg Engineering), which enables synchronous viewing of a selected location on an OCT scan image and an infrared fundus image. The process for classifying into *β*-zone and *γ*-zone PPA has been described in detail elsewhere [5]. In brief, *β*-zone PPA (based on conventional definition) was further classified into *β*-zone PPA with BM (*β*-zone group) and without BM (*γ*-zone group) on the basis of the location of the BM termination within the PPA area. The presence or absence of *β*-zone or *γ*-zone PPA in infrared fundus imaging and the classification of each type of PPA group were determined independently by two experienced ophthalmologists (E.H.J. and E.J.L.) who were masked to the clinical information of the patients. Disagreements were resolved by consensus between these two ophthalmologists or a third adjudicator (T.W.K.).

Since the purpose of this study was to determine whether the topographic relationship with the PMRE differs between *β*-zone and *γ*-zone PPA, eyes with the mixed type of PPA (i.e., having both *β*-zone and *γ*-zone PPA) were excluded from the analysis.

### 2.4. Topographic Measurements of RNFL Defects and the PMRE

**Figure 1:**
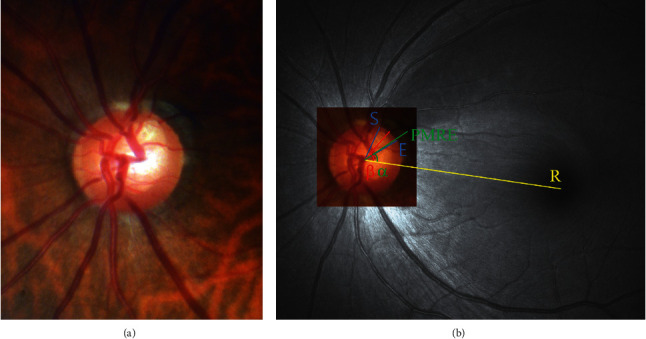
Measurement of the angular location of the point of maximum radial extent of parapapillary atrophy (PMRE) and a localized retinal nerve fiber layer (RNFL) defect and the angular extent of the localized RNFL defect. The PMRE and the RNFL defects were evaluated in a stereo disc photograph (a) and a red-free fundus photograph (b). The PMRE was the point on the parapapillary atrophy (PPA) margin at which the radial extent of PPA was maximal. The angular location of the PMRE was determined by measuring the angle between a reference line connecting the fovea and the disc center (line R [7, 13] and a line from the center of the disc to the PMRE (angle *α*)). Points S and E are the start and end points of the RNFL defect, respectively. The angular extent was defined as the circumferential angle between points S and E. The angular location was defined as the angle between line R and a line drawn from the center of the disc to the midpoint of the RNFL defect (angle *β*).

To quantify the topographic parameters of the RNFL defect, its angular location and extent were determined on red-free fundus photographs (Figure 1) [7]. A reference line was drawn from the center of the optic disc to the center of the fovea. The angular location of the RNFL defect was defined as the angle between the reference line and a line drawn from the center of the disc to the midpoint of the defect. The PMRE was defined as the point on the *β*-zone PPA margin where the radial extent of *β*-zone PPA was maximum [14]. The angular location of the PMRE was defined as the angle between the reference line and a line from the center of the disc to the PMRE. If the midpoint or PMRE was superior or inferior to the reference line, the location angle was designated as positive or negative, respectively. The angular extent of the RNFL defect was defined as a circumferential angle on the disc margin between the start and end points where the RNFL defect met the disc. In *γ*-zone PPA group, the angular extent of *γ*-zone PPA was additionally measured as a circumferential angle on the disc margin between the start and end points where *γ*-zone PPA met the disc.

The angular location of the RNFL defect and the PMRE and the extent of the RNFL defect and PPA were measured on a red-free fundus photograph using ImageJ (National Institutes of Health, Bethesda, MD, USA) by investigators who were masked to the clinical status of the patient. All measurements were made twice by two observers (E.H.J. and E.J.L.), and the mean value of the four measurement was used for the analyses.

### 2.5. Data Analysis

The intraobserver and interobserver agreements for measuring the topographic parameters of the RNFL defect and PPA were evaluated by calculating the intraclass correlation coefficient (ICC). Differences in continuous variables between two groups were compared using the independent *t*-test. Categorical variables were compared using the chi-square test. A Pearson correlation analysis was performed to assess the topographic correlation between the RNFL defect and PPA. All statistical analyses were performed with the Statistical Package for Social Sciences for Windows (version 22.0, SPSS, Chicago, IL, USA), and a *P* value less than 0.05 was accepted as significant.

## 3. Results

### 3.1. Baseline Characteristics

This study initially involved 259 eyes of 259 POAG patients who were enrolled in the IGPS and had a single localized RNFL defect. Of these, 170 patients were excluded due to the RNFL defect being <15° (*n* = 39) or >60° (*n* = 62), *β*-zone PPA being absent or narrower than 200 *μ*m (*n* = 31), or PPA being the mixed type (*n* = 38), leaving a final sample of 89 eyes of 89 patients. Of the 89 patients, 85 had IOP ≤21 mm Hg and 4 had IOP >21 mm Hg.

Table 1 compares the clinical characteristics between the *β*-zone and *γ*-zone groups. The age was significantly higher in the *β*-zone group (59.65 ± 9.62 years (mean ± standard deviation); range = 40–85 years) than in the *γ*-zone group (41.70 ± 9.22 years, range = 19–59 years; *P* < 0.001). There was no difference between the groups with respect to sex, untreated intraocular pressure (IOP), central corneal thickness, or visual field mean deviation (MD) and PSD. Systemic hypertension and diabetes mellitus were more prevalent in the *β*-zone than the *γ*-zone group. The eyes were significantly longer in the *γ*-zone group (26.17 ± 0.72 mm) than in the *β*-zone group (23.34 ± 0.78 mm, *P* < 0.001).

### 3.2. Group Comparisons of the Characteristics of the RNFL Defect and PPA

There was excellent interobserver agreement in measurements of the location and extent of the RNFL defect (ICC = 0.984 and 0.978, respectively) and of the location of the PMRE (ICC = 0.974). The intraobserver ICCs were 0.987 and 0.934 for the location and extent of the RNFL defect and 0.964 for the location of the PMRE. The maximum radial extent of PPA was larger in the *γ*-zone group (603.83 ± 257.81 *μ*m) than in the *β*-zone group (412.02 ± 125.85 *μ*m, *P* < 0.001).

**Figure 2:**
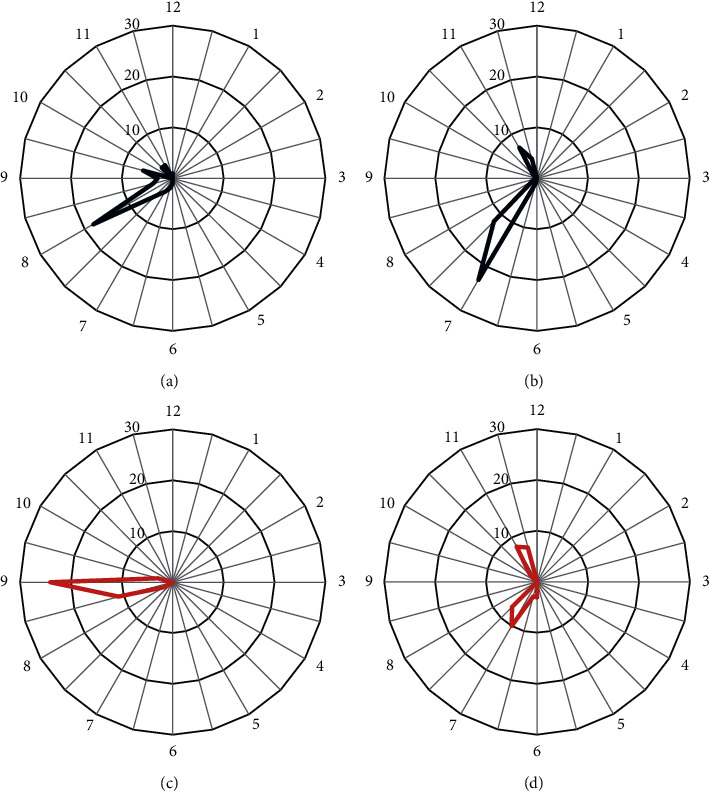
Histograms showing the frequency of the PMRE (a, c) and the RNFL defect location (b, d) in the *β*-zone and *γ*-zone groups, respectively. The PMRE and the RNFL defect locations were recorded based on the right eye orientation.

The angular extent of the RNFL defect did not differ between the *β*-zone and *γ*-zone PPA groups. The RNFL defect was most often found in the *β*-zone group in the inferotemporal sector (75.5%), followed by the superotemporal sector (24.5%). The inferotemporal sector was also the most common site of the RNFL defect in the *γ*-zone group. However, the frequency of the RNFL defect in the superotemporal sector was comparable to that in the inferotemporal sector (42.5% vs. 57.5%, Figure 2). The frequency difference in the hemispheric location of the RNFL defect was borderline significant between the groups (*P*=0.071, Table 2).

**Figure 3:**
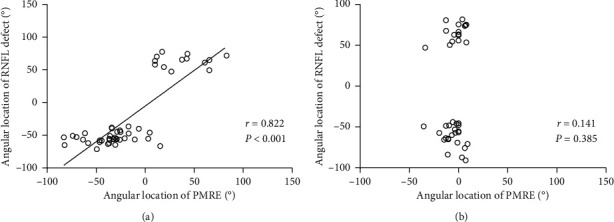
Scatter plots showing the relationship between the angular location of the PMRE of PPA and that of the RNFL defect in *β*-zone PPA (a) and *γ*-zone PPA (b).

**Figure 4:**
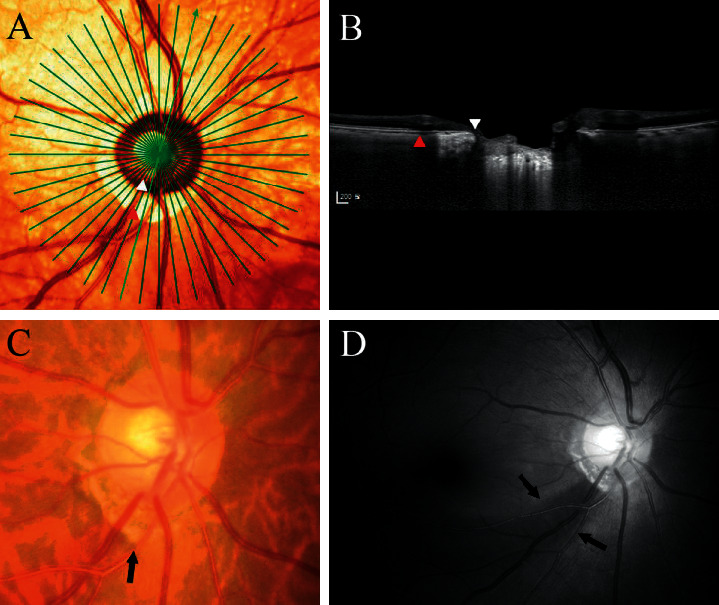
Representative case showing the topographic relationship between the PMRE and the RNFL defect in an eye with *β*-zone PPA. (a) Pseudocolor infrared fundus photograph showing the 24 radial B-scan lines. (b) Optical coherence tomography (OCT) image showing *β*-zone PPA. White and red arrowheads indicate the optic disc margin and PPA margin, respectively. Bruch's membrane (BM) is seen throughout the PPA bed. (c) The PMRE is seen near the 6.5-o'clock meridian (arrow). (d) The RNFL defect (arrows) is seen near the PMRE.

**Figure 5:**
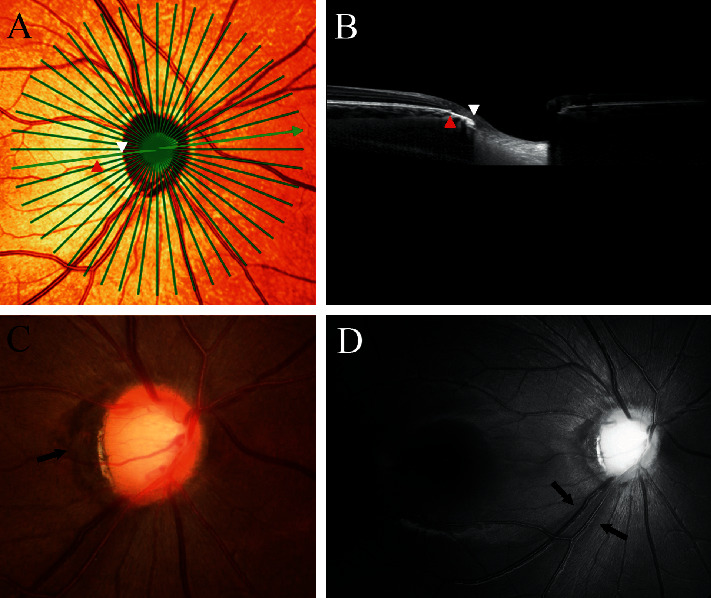
Representative case showing the topographic relationship between the PMRE and the RNFL defect in an eye with *γ*-zone PPA. (a) Pseudocolor infrared fundus photograph showing the 24 radial B-scan lines. (b) Radial B-scan OCT image showing *γ*-zone PPA. White and red arrowheads indicate the optic disc margin and PPA margin, respectively. BM is lacking throughout the PPA bed. (c) The PMRE is seen near the 8.5-o'clock meridian (arrow). (d) The RNFL defect (arrows) is seen near the edge of PPA.

**Figure 6:**
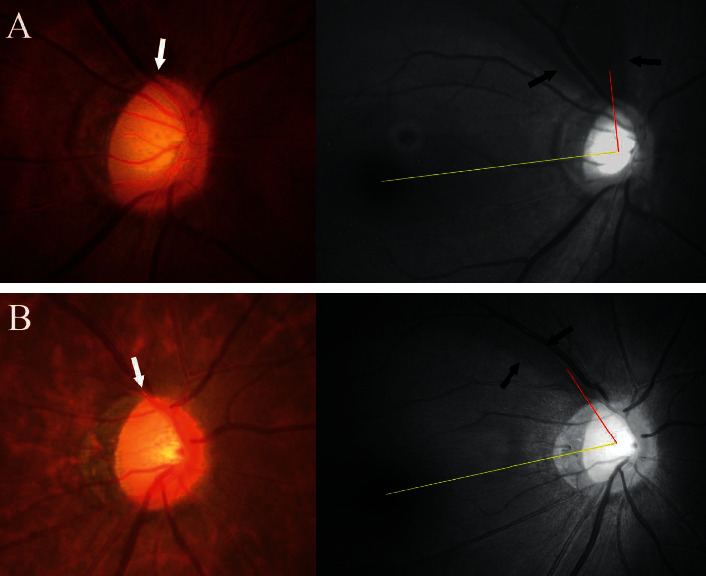
Cases showing the presence of superior retinal nerve fiber layer (RNFL) defect at the edge of parapapillary atrophy (PPA) in eyes with *γ*-zone PPA. Color disc photographs are in the left. Arrows indicate the location of superior PPA edge. Red-free photographs are in the right. Note that the optic disc is slightly torted superiorly in (a) and inferiorly in (b). The angle between the fovea-disc center axis (yellow line) and the line drawn from the center of the disc to the midpoint of the RNFL defect (red line) is considerably different between the two eyes. Note that the angular distance of the superiorly located RNFL defect is greater in A which had a superior torsion than B which had a slight inferior torsion.

**Figure 7:**
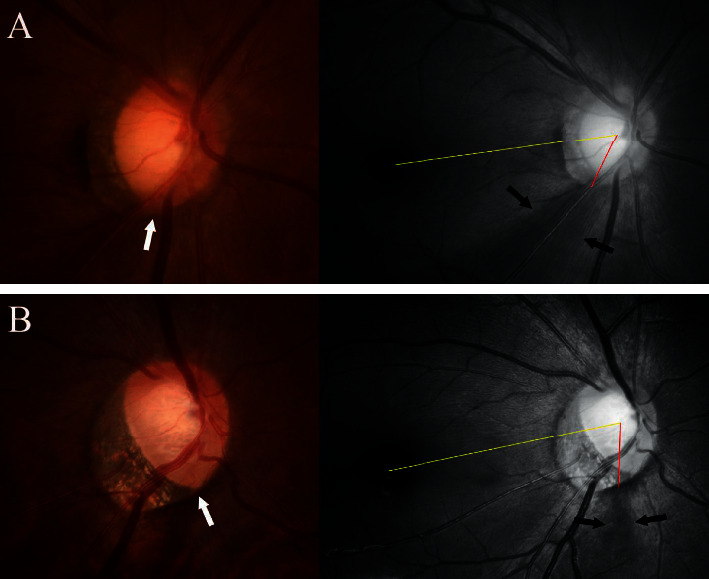
Cases showing the presence of inferior retinal nerve fiber layer (RNFL) defect at the edge of parapapillary atrophy (PPA) in eyes with *γ*-zone PPA. Color disc photographs are in the left. Arrows indicate the location of inferior PPA edge. Red-free photographs are in the right. Note that the optic disc is considerably torted inferiorly in (b). The angle between the fovea-disc center axis (yellow line) and the line drawn from the center of the disc to the midpoint of the RNFL defect (red line) is considerably different between the two eyes. Note that the angular distance of the inferiorly located RNFL defect is greater in B which had greater inferior torsion than (a).

In the *β*-zone group, the angular location of the RNFL defect was significantly associated with that of the PMRE (*r* = 0.822, *P* < 0.001). However, no such relationship was found in the *γ*-zone group (*r* = 0.141, *P*=0.385; Figures 2 and 3). In addition, the angular distance between the RNFL defect and the PMRE differed significantly between *β*-zone and *γ*-zone PPA (26.49 ± 17.27° vs. 60.31 ± 17.12°, *P* < 0.001; Table 2). The RNFL defect was mostly located near the edge of PPA in eyes with *γ*-zone PPA. The angular distance between the RNFL defect and the closer edge of *γ*-zone PPA was 10.56 ± 9.47°. Accordingly, there was a strong topographic correlation between the edge of *γ*-zone PPA and the RNFL defect (*r* = 0.983, *P* < 0.001). Representative cases showing the relationship between the PMRE and the RNFL defect in eyes with *β*-zone and *γ*-zone PPA are shown in Figures 4 and 5, respectively. Figures 6 and 7 shows the RNFL defects observed near the superior and inferior edge of *γ*-zone PPA, respectively.

## 4. Discussion

We have evaluated the topographic relationship of the RNFL defect with the PMRE in POAG eyes with *β*-zone and *γ*-zone PPA. The location of the RNFL defect was significantly correlated with the PMRE in eyes with *β*-zone PPA, which is consistent with a previous report [7]. In contrast, no such a relationship was found in eyes with *γ*-zone PPA, whereas the RNFL defect was found mostly at the edge of PPA. To the best of our knowledge, there is no report in the literature comparing the spatial relationship between the PMRE and the RNFL defect between glaucomatous eyes with *β*-zone and *γ*-zone PPA.

The current study included only eyes with single localized RNFL defect. This was because it is reasonable to analyze the earliest RNFL loss in order to evaluate the influence or relevance (not necessarily implicating that PPA preceded RNFL defect) of PPA on the development location of an RNFL defect. It is difficult to determine the location of the first RNFL damage in late-stage glaucoma with a large-extent diffuse RNFL defect and multiple localized RNFL defects.

It is noteworthy that patients in the *γ*-zone group were markedly younger than those in the *β*-zone group, despite having similar angular extents of the RNFL defect and visual field MD. This finding suggests that glaucomatous optic neuropathy develops earlier in eyes with *γ*-zone PPA. Studies have demonstrated that *γ*-zone PPA is often found in young patients with myopia and associated with externally oblique border tissue of Elschnig [5, 6]. These data suggest that *γ*-zone PPA results from scleral stretching associated with elongation of the eyeball at an early age. Indeed, the development of *γ*-zone PPA together with progressive optic disc shape change has been documented in children with myopic shift [8, 9]. According to this paradigm, the eyes with *γ*-zone PPA are subject to mechanical stress arising from scleral stretching [9]. This may cause distortion of the optic nerve structure at an early age, particularly in the lamina cribrosa [15, 16], and exert a tensile or shearing stress on the axons passing through the lamina pores. Long-standing stress from an early age could generate axonal damage by itself [17] or increase the susceptibility of the axons to IOP-related mechanical stress [18]. This could explain why eyes with *γ*-zone PPA have an RNFL defect of similar extent at an earlier age compared to those with *β*-zone PPA.

The choroidal and deep ONH structures including prelaminar tissue and the lamina cribrosa are both supplied by the posterior ciliary artery. In addition, the prelaminar area is at least partly supplied by the centripetal branches of the peripapillary choroidal arterioles [19]. Histologic studies demonstrate diminution or loss of the choriocapillaris and a decreased choroidal thickness in *β*-zone PPA [20–22]. In accordance with this, angiographic studies have also demonstrated decreased choroidal perfusion in eyes with *β*-zone PPA [23, 24]. Together these findings suggest that vascular perfusion to the prelaminar area is disturbed in eyes with *β*-zone PPA. Given that axonal transport requires a large amount of energy [25, 26], the resulting vascular insufficiency would hamper the axonal transport in the involved region. This process may induce retinal ganglion cell (RGC) death by itself or in conjunction with IOP-related stress [27]. The topographic association between the PMRE and the RNFL defect is consistent with this concept. It is reasonable to expect that the negative influence on the intrapapillary perfusion is maximal at or near the location of the PMRE.

In contrast, the PMRE and RNFL defects were far apart in eyes with *γ*-zone PPA, with the RNFL defect observed near the edge of PPA in these eyes. The mechanism underlying this intriguing finding is unclear. It is possible that the development of the RNFL defect at the edge of the *γ*-zone is simply coincidental. The superotemporal and inferotemporal sectors are known to be preferentially involved in glaucoma due to the anatomic connective tissue support being lowest at the level of the lamina cribrosa in this area. Since *γ*-zone PPA develops mostly on the temporal side of the optic disc, the edge of the *γ*-zone would be located in the superotemporal and inferotemporal sectors, and so near the most frequent location of glaucomatous RNFL defects. However, we consider this possibility less likely based on RNFL defects frequently occurring at earlier ages and being often located in the superotemporal sector in the *γ*-zone group. If the RNFL defects developed via the same mechanisms as in eyes without *γ*-zone PPA, those differing features would not be seen. Moreover, the RNFL defects were not simply found in the superotemporal and inferotemporal sectors, but their location varied in accordance with the location of the edge of *γ*-zone PPA (Figures 6 and 7).

As discussed earlier, it is considered that *γ*-zone PPA is derived from scleral stretching arising from the axial elongation of the eyeball. Since the lamina cribrosa is anchored to the peripapillary sclera, the stress from scleral stretching may induce structural lateration in the lamina cribrosa. In addition, the stretch growth of the sclera associated with axial elongation may induce tensile stress to the axons. The preferential location of a RNFL defect near the edge of *γ*-zone PPA suggests that a meaningful stress arising from axial elongation is concentrated to the lamina cribrosa or axons near the edge of *γ*-zone PPA. The mechanism that explains this hypothesis is currently unclear. Further studies elucidating the mechanism underlying the preferential location of the RNFL defect near the edge of the *γ*-zone may help understand the biomechanics of glaucomatous optic nerve damage in myopic tilted eyes.

Kimura et al. [28] reported that papillomacular bundle defects are common in highly myopic eyes (refractive error < −6 diopters) with early glaucoma. Those authors suggested that this can be attributed to the increased tensile stress on the temporal side of the lamina cribrosa and the RGC axons in the papillomacular bundle. Given that *γ*-zone PPA probably results from scleral stretching and the PMRE is generally located near the papillomacular bundle in eyes with *γ*-zone PPA, it may be expected that papillomacular bundle defects would be often seen in patients with *γ*-zone PPA. However, such defects were not found in the *γ*-zone group in the present study, and we attribute this discrepancy mainly to the inclusion of patients with only a single RNFL defect. All of the presented representative cases of papillomacular bundle defect in the study of Kimura et al. [28] had multiple RNFL defects. In addition, many of our patients had mild or moderate myopia, whereas Kimura et al. included highly myopic eyes exclusively. It is possible that a papillomacular bundle defect often occurs in highly myopic eyes but not as the earliest (first) RNFL defect.

Glaucoma is currently treated by lowering the IOP [29–31]. The natural disease history and the disease course after IOP-lowering treatment vary markedly among patients [2, 3, 32–35]. This highlights the need to subclassify the patients based on the underlying pathogenic mechanism and to determine the natural course and/or response to treatment in each subgroup [36–38]. Studies have demonstrated that progression is slower in patients with *γ*-zone PPA than in those with *β*-zone PPA and those without PPA [39, 40]. Therefore, elucidating the characteristics of the RNFL damage and knowing the mechanism underlying the damage in eyes with *γ*-zone PPA is important for the better management of patients with myopic glaucoma. The preferential location of the RNFL defect in eyes with *γ*-zone PPA (i.e., the edge of *γ*-zone PPA) needs to be considered in research studies targeting the pathogenesis and clinical course of myopic glaucoma.

This study was subject to some limitations. First, we speculated that *β*-zone and *γ*-zone group may have a different pathogenesis based on their association with differing covariate factors. Second, since the purpose of the study was to determine whether the topographic relationship with the PMRE differed between *β*-zone and *γ*-zone PPA, eyes with the mixed type of PPA (i.e., *β*-zone and *γ*-zone) were excluded. Further study is needed on the topography between RNFL defects and PPA in those eyes. We also excluded eyes with degenerative myopia, so our results might not be applicable to eyes with high degenerative myopia. Third, the number of eyes in *β*-zone and *γ*-zone group was relatively small. However, absence of correlation between the RNFL defect and PMRE of *γ*-zone was also found when tested nonparametrically using Spearman's rank correlation coefficient analysis. Last, the subjects were all Korean, so it remains to be determined whether the present findings also apply to other races.

In conclusion, RNFL defects were observed near the edge of PPA in eyes with *γ*-zone PPA, whereas it was spatially related with the PMRE in eyes with *β*-zone PPA, suggesting that a greater pathogenically meaningful stress is given to the axons near the edge of *γ*-zone PPA than in the central PMRE. Further study is needed to explore the underlying mechanism for the preferential location of RNFL defects near the edge of *γ*-zone PPA.

## Figures and Tables

**Table 1:** Comparison of variables between *β*-zone and *γ*-zone parapapillary atrophy group.

	*β*-zone group (49 eyes)	*γ*-zone group (40 eyes)	*P* value
Age (year)	59.65 ± 9.62	41.70 ± 9.22	**<0.001** ^*∗*^
Female, *n* (%)	24 (49.0)	16 (40.0)	0.397^†^
Hypertension, *n* (%)	16 (32.7)	5 (12.5)	**0.026** ^**†**^
Diabetic mellitus, *n* (%)	12 (24.5)	2 (5.0)	**0.012** ^**†**^
Cold hands, *n* (%)	11 (28.2)	7 (18.4)	0.310^†^
Untreated intraocular pressure (mm Hg)	15.27 ± 3.04	15.65 ± 3.33	0.571^*∗*^
Refractive error (D)	−0.08 ± 1.49	−5.60 ± 1.84	**<0.001** ^*∗*^
Axial length (mm)	23.34 ± 0.78	26.17 ± 0.72	**<0.001** ^*∗*^
Central corneal thickness (*μ*m)	563.33 ± 38.50	554.88 ± 34.68	0.377^*∗*^
Visual field (dB)			
MD	−3.06 ± 2.28	−2.78 ± 2.64	0.594^*∗*^
PSD	5.45 ± 3.67	4.76 ± 3.71	0.378^*∗*^

Data are presented as mean ± standard deviation or number (%). ^*∗*^Comparison performed using independent *t*-test. ^†^Comparison performed using chi-square test.

**Table 2:** Comparison of locational correlation with retinal nerve fiber layer defect between *β* zone and *γ*-zone parapapillary atrophy group.

	*β*-zone group (49 eyes)	*γ*-zone group (40 eyes)	*P* value
Angular extent of RNFL defect (°)	29.88 ± 11.65	30.14 ± 12.44	0.920^*∗*^
Location of RNFL defect, *n* (%)			0.071^†^
Superior	12 (24.5)	17 (42.5)	
Inferior	37 (75.5)	23 (57.5)	
Angular location of RNFL defect (°)	−24.77 ± 52.26	−7.37 ± 64.38	0.172^*∗*^
Angular location of PMRE (°)	−15.86 ± 38.38	−4.44 ± 10.06	0.050^*∗*^
Width of PPA on PMRE (*μ*m)	412.02 ± 125.85	603.83 ± 257.81	**<0.001** ^*∗*^
The angular distance between the RNFL defect and PMRE (°)	26.49 ± 17.27	60.31 ± 17.12	**<0.001** ^*∗*^

RNFL = retinal nerve fiber layer, PMRE = point of maximum radial extent on the parapapillary atrophy margin, PPA = parapapillary atrophy. Data are presented as mean ± standard deviation. ^*∗*^Comparison performed using independent *t*-test. ^†^Comparison performed using chi-square test.

## Data Availability

The data sets generated and analyzed for the current study are available from the corresponding author upon reasonable request.
